# ﻿Complete mitochondrial genome of *Plecialongiforceps* Duda, 1933 (Diptera, Bibionidae) and its implications for a phylogeny of the family Bibionidae

**DOI:** 10.3897/zookeys.1212.117845

**Published:** 2024-09-12

**Authors:** Seunghun Jung, Sangil Kim, Seunggwan Shin

**Affiliations:** 1 School of Biological Sciences, Seoul National University, Seoul 08826, Republic of Korea; 2 Research Institute of Basic Sciences, Seoul National University, Seoul 08826, Republic of Korea; 3 Museum of Comparative Zoology and Department of Organismic and Evolutionary Biology, Harvard University, Cambridge, MA 02138, USA

**Keywords:** Bibionidae, Bibionomorpha, invasive species, long-read sequencing, mitogenome, phylogenetic analysis

## Abstract

Over the past decade, the prevalence of mass outbreaks involving non-native insects has sparked concerns about their potential negative impact on human inhabited areas and local environments. *Plecialongiforceps* Duda, 1933 (Diptera, Bibionidae) was recently recognized as an invasive pest in South Korea, causing public nuisance through mass outbreaks in the Seoul Metropolitan Area during early summer. In this study, we present the first complete mitochondrial genome of *Plecialongiforceps*, generated from the PacBio HiFi long-read sequencing data. Notably, the length of the circular genome is found to be larger than any annotated reference sequences of mitochondrial genomes for the infraorder Bibionomorpha, which is attributable to an unusually long A+T rich control region. We conducted a phylogenetic analysis of Bibionomorpha, focusing specifically on the family Bibionidae, using nearly all available mitochondrial genome data to elucidate relationships among genera within Bibionidae. Our phylogeny of Bibionomorpha recovered a strong monophyly of the family Bibionidae and its three subfamilies: Bibioninae (*Bibio* + *Dilophus*), Hesperininae (*Hesperinus* + *Penthetria*), and Pleciinae (*Plecia*), corroborating the recently proposed taxonomic classification system of Bibionidae. Furthermore, we discuss evolutionary trends within Bibionidae based on our well-supported higher relationships of the superfamily Bibionoidea.

## ﻿Introduction

The infraorder Bibionomorpha is a species-rich group of the insect order Diptera, comprising of approximately 15,000 described extant species worldwide (Evenhuis and Pape 2023). These nematoceran flies are renowned for their remarkable morphological and ecological adaptations to diverse habitats, which is reflected in their biodiversity. Bibionomorpha are considered closely related to the suborder Brachycera, with both groups collectively called Neodiptera, rendering Nematocera as a paraphyletic grade (Amorim and Yeates 2006). The Mesozoic subfamily †Burmahesperininae (Diptera, Bibionidae) described from the Mid-Cretaceous amber of Myanmar presents wing venation resembling that of the genus *Hesperinus* Walker, 1848, but with brachyceran-like modifications of the antennae. These modifications include antennal segments differentiated into three parts, each with a different number of flagellomeres – the basal segment as scape, the second segment as pedicel, and the third segment as stylus – potentially supporting the concept of Neodiptera (Ševčík et al. 2021). While most species of Bibionomorpha show little association with human activities, certain groups hold economic significance as pests. For instance, the larvae of gall midges (Diptera, Cecidomyiidae) and dark-winged fungus gnats (Diptera, Sciaridae) are widely recognized for occasionally damaging crops and mushroom cultivation. Synchronized mass emergence of several members of the family Bibionidae with terrestrial and saprophagous larvae have historically been reported during the spring to late summer. In particular, this mass emergence with swarming of adult bibionids is considered a public nuisance and is increasing in both frequency and geographical range in recent years, indicating their potential expansion into urban environments due to anthropogenic influence and climate change (Qvenild and Rognerud 2017; Krivosheina et al. 2019; Abou-Shaara et al. 2022). Therefore, understanding the nature of insect outbreaks has increasingly become important for pest control and prevention through the regulation of factors associated with population dynamics of target species.

*Plecialongiforceps* Duda, 1933 (Diptera, Bibionidae) is an invasive pest in East Asia, characterized by its seasonal mass outbreak of adult mating pairs near suburban areas (Fitzgerald and Nakamura 2015; Kim et al. 2022). The recent introduction of this species to the Korean Peninsula caused public disturbance since the summer of 2022, and such occurrences are expected to become increasingly prevalent, favoring the trend of climate changes within the north temperate zone (Kim et al. 2022). This case parallels the classic example of the so-called “lovebug” or “march flies”, *Plecianearctica* Hardy, 1940, in the southeastern United States, with its Central American origin and gradual expansion history into North America through the 20^th^ century (Hardy 1940; Buschman 1976). Despite the obvious importance, knowledge of the evolutionary history and biology of the genus *Plecia* Wiedemann, 1828, along with other bibionids, remain unsatisfactory, hindering the assessment of their ecological impacts and prediction of future outbreaks. Traditionally, Hardy and Takahashi (1960) considered the genus *Plecia* to be closely related to the genus *Penthetria* Meigen, 1803 and treated them as genera within the subfamily Pleciinae. Later cladistic studies, focusing on extant species, found the Pleciinae concept of *Plecia* + *Penthetria* to be paraphyletic and treated *Plecia* as the sole member of Pleciinae, sister to the subfamily Bibioninae (Fitzgerald 2004) – i.e., the four-subfamily system proposed by Pinto and Amorim (2000). The recent phylogenies of Bibionidae based on molecular (Ševčík et al. 2016) and morphological evidence (Skartveit 2009; Skartveit and Ansorge 2020) supported the three-subfamily system of Bibionidae that includes a monophyletic group of the subfamily Hesperininae Schiner, 1868, consisting of *Hesperinus* Walker, 1848, and *Penthetria* Meigen, 1803. However, the synapomorphies supporting this relationship have not been firmly established.

In recent years, with increased accessibility to next-generation sequencing technologies, genomic data for various insects have been generated rapidly. A recent study of 16 newly sequenced bibionomorphan species emphasized the evolution of gene-rich X chromosomes and sex determination systems in nematoceran flies, expanding our knowledge of genomic evolution to previously neglected groups of Bibionomorpha, such as the families Anisopodidae and Bibionidae (Anderson et al. 2022). In addition, chromosomal-level genome assemblies for two species of Bibionidae, *Bibiomarci* (Sivell et al. 2021) and *Dilophusfebrilis* (GenBank accession: GCA_958336335.1), provide invaluable resources for future genomic studies of this group. However, the majority of available genome sequencing data are focused on mitochondrial sequences, which is largely attributable to the practicality of using DNA markers derived from mitochondrial genomes (mitogenome) in DNA-based species identification and phylogenetic analysis (Cameron 2014). The advent of long-read sequencing technologies has allowed for an efficient and robust assembly and annotation of complete mitogenomes, providing opportunities for comparative studies of previously neglected features, such as tandem repeats in the control region. This advancement overcomes technological constraints associated with short-read sequencing (Morgan et al. 2022), which is prone to assembly errors in repeat-rich regions. The application and utility of resolving deep relationships within Diptera using mitogenomes have been extensively tested over a decade (Cameron et al. 2007), including Bibionomorpha. Previous studies have predominantly focused on resolving relationships within nematoceran lineages based on mitogenomes (Beckenbach 2012, Xiao et al. 2023, Zhang et al. 2023). Despite the significance of this group in addressing the phylogenetic gap between “lower flies” (Nematocera) and more derived “higher flies” (Brachycera), a comprehensive analysis of all extant subfamilies of Bibionidae based on mitochondrial genomic data is yet to be conducted.

In this study, we present the first complete and annotated mitogenome of *Plecialongiforceps* to provide insights into the phylogeny and evolutionary trends of Bibionidae from a mitochondrial genomic perspective. Furthermore, we manually annotated mitochondrial genes from previously available mitogenome data or whole-genome assemblies of Bibionomorpha and one species of Axymyiidae (Diptera, Axymyiomorpha). We then incorporated these genes into the phylogenetic analysis to reconstruct the evolutionary history at the infraorder level.

## ﻿Materials and methods

### ﻿Sample collection, DNA extraction, and sequencing

We collected a larval specimen of *Plecialongiforceps* from Incheon (37°23.34'N, 126°43.24'E), South Korea, in April 2023. After the adult emerged from its pupa, we performed identification based on the morphology of adult male terminalia following the previous studies (Fitzgerald and Nakamura 2015; Kim et al. 2022). Subsequently, we flash froze the specimen in liquid nitrogen and stored it at −80 °C until genomic DNA extraction.

The high molecular weight (HMW) genomic DNA (gDNA) was extracted from a single adult specimen using a modified version of the cetrimonium bromide (CTAB) precipitation method. To ensure the high molecular weight of the extracted gDNA, we assessed the gDNA extract through gel electrophoresis on a 1% agarose gel with the lambda DNA marker, and we quantified it with the Quantus Fluorometer (Promega, USA) and Nanodrop Spectrophotometer (Thermo Fisher Scientific, USA). Additionally, we treated the purified HMWgDNA with the Short Read Eliminator (SRE) XL kit (Pacific Biosciences, USA) to remove short DNA fragments below 40 kb and then sheared it into 20 kb fragments using the Megaruptor 2 (Diagenode, Belgium). We constructed the PacBio SMRT library using the SMRTbell Prep Kit 3.0 and sequenced it on a single SMRT HiFi cell of the PacBio Sequel IIe system (Pacific Biosciences, USA) at the National Instrumentation Center for Environmental Management (NICEM), Seoul National University (Seoul, Republic of Korea). Finally, we processed the raw base-called data through the Circular Consensus Sequence analysis application of SMRT® Link (ver. 12.0.0.177059) to identify high fidelity (HiFi) reads.

### ﻿Mitochondrial genome assembly and annotation

From the newly produced PacBio HiFi reads, we isolated the mitochondrial reads and assembled them using the MitoHiFi pipeline v. 3.2 (Uliano-Silva et al. 2023), with *Bradysiaodoriphaga* Yang & Zhang, 1985 (GenBank accession: NC_061662.1) as the reference sequence. Among the candidate contigs, we chose a single representative mitochondrial contig based on the annotation result obtained from MitoFinder (Allio et al. 2020). Manual curation and annotation of non-coding regions were performed in Geneious Prime® 2023.2.1. The online version of Proksee (Grant et al. 2023) was used for the calculation of GC content and GC skew, and for preparing a structure map in a JSON file. A circular map of the mitogenome with gene elements was drawn using the CGview server (Grant and Stothard 2008). Furthermore, unannotated mitogenomes of four bibionomorphs (*Bibiomarci*, *Bibiorufiventris*, *Dilophusfebrilis*, and *Plecia* sp.), one species of the family Axymyiidae (*Protaxymyia* sp.), and four whole genome assemblies of bibionomorphs (*Bolitophilahybrida*, *Diadocidiaferruginosa*, *Penthetriafunebris*, and *Symmerusnobilis*) were retrieved from NCBI GenBank (as of October 2023) and underwent processing using the same annotation methodology that was applied to our newly assembled mitogenome (Table 1).

**Table 1.** Information on the mitochondrial genome data used in the present study. GenBank accession number of the manually annotated taxa are highlighted in bold, and those newly assembled and annotated are marked with an asterisk.

Suborder/Infraorder	Family	Species	GenBank accession numbers	Reference
Axymyiomorpha	Axymyiidae	*Protaxymyia* sp.	**MZ562679.1**	Zhang et al. (2023)
Bibionomorpha	Anisopodidae	*Sylvicolafenestralis* (Scopoli, 1763)	NC_016176.1	Beckenbach (2012)
Bibionomorpha	Bibionidae	*Bibiomarci* (Linnaeus, 1758)	**OU343120.2**	Sivell et al. (2021)
Bibionomorpha	Bibionidae	*Bibiorufiventris* Duda, 1930	MZ562678.1	Zhang et al. (2023)
Bibionomorpha	Bibionidae	*Dilophusfebrilis* (Linnaeus, 1758)	**OY284474.1**	Unpublished
Bibionomorpha	Bibionidae	*Hesperinusbrevifrons* Walker, 1848	See Table 2	Ševčík et al. (2016)
Bibionomorpha	Bibionidae	*Hesperinusninae* Papp & Krivosheina, 2009	See Table 2	Ševčík et al. (2016)
Bibionomorpha	Bibionidae	*Penthetriafunebris* Meigen, 1804	GCA_027564355.1*	Anderson et al. (2022)
Bibionomorpha	Bibionidae	*Plecialongiforceps* Duda, 1933	PP060435*	Present study
Bibionomorpha	Bibionidae	*Plecia* sp.	**MZ562680.1**	Zhang et al. (2023)
Bibionomorpha	Bolitophilidae	*Bolitophilahybrida* (Meigen, 1804)	GCA_027564075.1*	Anderson et al. (2022)
Bibionomorpha	Cecidomyiidae	*Mayetioladestructor* (Say, 1817)	GQ387648.1	Beckenbach and Joy (2009)
Bibionomorpha	Cecidomyiidae	*Orseoliaoryzae* (Wood-Mason, 1889)	KM888183.1	Atray et al. (2015)
Bibionomorpha	Diadocidiidae	*Diadocidiaferruginosa* (Meigen, 1830)	GCA_027564275.1*	Anderson et al. (2022)
Bibionomorpha	Ditomyiidae	*Symmerusnobilis* Lackschewitz, 1937	GCA_027564815.1*	Anderson et al. (2022)
Bibionomorpha	Keroplatidae	*Arachnocampaflava* Harrison, 1966	NC_016204.1	Beckenbach (2012)
Bibionomorpha	Keroplatidae	*Orfelia* sp.	**MW394227.1**	Zhang et al. (2023)
Bibionomorpha	Mycetophilidae	*Acnemianitidicollis* (Meigen, 1818)	NC_050318.1	Unpublished
Bibionomorpha	Mycetophilidae	*Allodiaprotenta* Laštovka & Matile, 1974	NC_060624.1	Unpublished
Bibionomorpha	Pachyneuridae	*Cramptonomyiaspenceri* Alexander, 1931	NC_016203.1	Beckenbach (2012)
Bibionomorpha	Sciaridae	*Bradysiaodoriphaga* Yang & Zhang, 1985	NC_061662.1	Unpublished
Bibionomorpha	Sciaridae	*Sciararuficauda* Meigen, 1818	NC_046767.1	Miao et al. (2020)
Brachycera	Dolichopodidae	*Dolichopusgaleatus* Loew, 1871	NC_070101.1	Wang et al. (2023)
Brachycera	Stratiomyidae	*Parastratiosphecomyiaszechuanensis* Lindner, 1954	NC_053880.1	Hu and Yang (2021)
Brachycera	Tabanidae	*Tabanuschrysurus* Loew, 1858	NC_062705.1	Unpublished
Psychodomorpha	Scatopsidae	*Coboldiafuscipes* (Meigen, 1830)	MZ567016.1	Zhang et al. (2023)
Tipulomorpha	Tipulidae	*Tipulaaestiva* Savchenko, 1960	NC_063751.1	Unpublished

### ﻿Phylogenetic analysis

We employed both maximum-likelihood (ML) and Bayesian inference (BI) methods to infer phylogenetic trees. Given the sparse availability of mitogenomic data for Bibionoidea compared to Sciaroidea, we included two representative taxa for each recognized family within Sciaroidea to mitigate the potential bias that may arise from uneven taxon sampling between across the groups. Our final dataset included a total of 27 terminal taxa, encompassing mitogenomes from 25 representative dipteran species and partial sequences of the 12S, 16S ribosomal RNA genes and cytochrome oxidase *c* subunit I (COI) from two *Hesperinus* species – *Hesperinusbrevifrons* and *Hesperinusninae*. These sequences were derived from Ševčík et al. (2016), with the exception of the COI sequence for *H.brevifrons*, which was downloaded from the National Center for Biotechnology Information (NCBI) genetic sequence database (GenBank accession: JN294723.1). As outgroup taxa, three non-bibionomorphan species of the suborder Nematocera (*Coboldiafuscipes*, *Tipulaaestiva*, and *Protaxymyia* sp.) and three species of the suborder Brachycera (*Dolichopusgaleatus*, *Parastratiosphecomyiaszechuanensis*, and *Tabanuschrysurus*) were incorporated (Tables 1, 2). We aligned the sequences of 13 protein-coding and two ribosomal RNA genes of mitogenomes using the command-line version of MAFFT v. 7.475 with default parameters (Katoh and Standley 2013). The concatenated matrix of gene alignments was generated using the ‘create_concatenation_matrix’ function implemented in PhyKIT (Steenwyk et al. 2021). Best-fitting nucleotide substitution models were determined for each gene alignment using PartitionFinder2 (Lanfear et al. 2017) under the corrected Akaike Information Criterion (AICc). Data blocks for the protein-coding genes were pre-defined to reflect all three codon positions. The ML analysis was conducted in IQ-TREE v. 2.2.2.6 (Minh et al. 2020) using the partition mode, and branch support values assessed via ultra-bootstrap approximation method with 5,000 bootstrap replicates. The BI analysis was carried out in MrBayes v. 3.2.7a (Ronquist et al. 2012), running four chains for 10 million generations with trees sampled every 10,000 generations. Convergence of the runs was diagnosed in Tracer v. 1.7.2 (Rambaut et al. 2018) with the first 2.5 million generations discarded as burn-in.

**Table 2.** GenBank accession information on the three mitochondrial genes for the two *Hesperinus* species used in this study.

Taxon	12S	16S	COI
*Hesperinusbrevifrons* Walker, 1848	KP288705.1	KP288737.1	JN294723.1
*Hesperinusninae* Papp & Krivosheina, 2009	KP288687.1	KP288719.1	KT316856.1

## ﻿Results and discussion

### ﻿General mitochondrial genomic characteristics

**Figure 1.**
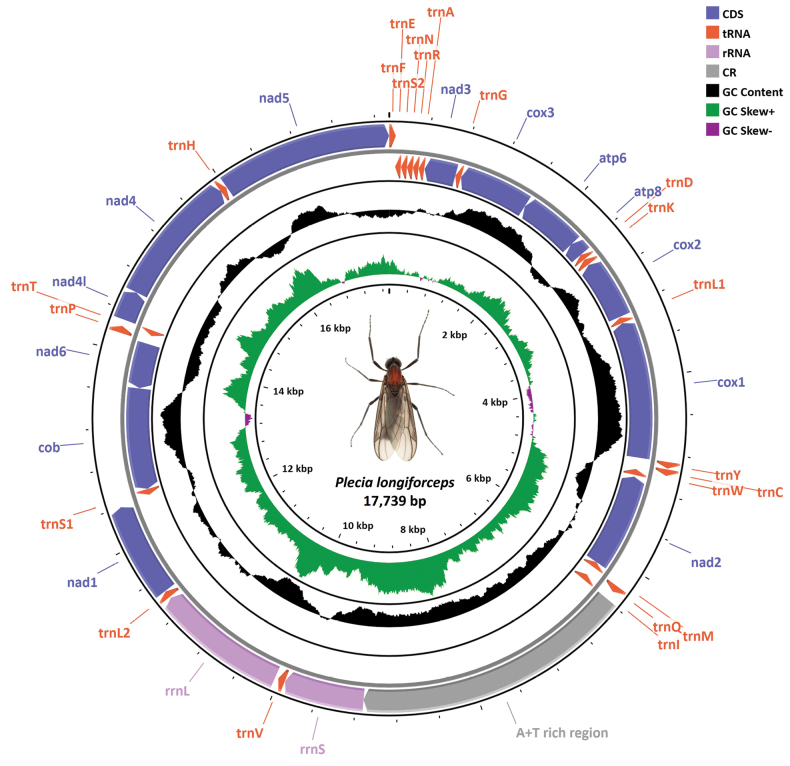
Circular map of the newly sequenced and assembled mitochondrial genome of *Plecialongiforceps* with a ha,bitus of adult male specimen in dorsal view at center. All protein coding genes, ribosomal RNA genes, transfer RNA genes and a control region are shown with the feature table definition from the International Nucleotide Sequence Database Collaboration (INSDC). The direction of gene transcription is indicated with an arrow. The length of each gene is proportional to its nucleotide length. The innermost and middle circles indicate the GC skew and GC content, respectively. The outermost circle displays gene arrangement. Abbreviations: CDS, coding sequences; rRNA, ribosomal RNA; tRNA, transfer RNA; CR, control region.

Using the PacBio HiFi long-read sequencing technology, we successfully sequenced and assembled the complete mitochondrial genome (mitogenome) of *Plecialongiforceps*. Our initial PacBio HiFi data yielded a total of 27,997,014,611 base pairs (bp) in 1,891,452 reads, of which MitoHiFi identified only 20 reads as mitochondrial. The assembled mitogenome of *P.longiforceps* is circular, measuring 17,739 bp in length, with a base composition of 42.0% A, 39.6% T, 7.5% C, and 10.8% G. The annotation result comprised of 37 genes – 13 protein-coding genes, 2 ribosomal RNA genes, and 22 transfer RNA genes – along with one A+T rich control region (Fig. 1), consistent with the putative ancestral insect mitogenome in gene contents and arrangements (Cameron 2014). These highly conserved aspects of the *P.longiforceps* mitogenome are readily observed in other bibionomorph lineages, with a few exceptions reported in families such as Cecidomyiidae and Keroplatidae (Xiao et al. 2023). Notably, our mitogenome assembly of *P.longiforceps* is significantly longer than that of *Plecia* sp. (GenBank ﻿accession: MZ562680.1; 15,763 bp), despite the overall similarity between the two mitogenomes in terms of gene contents and respective nucleotide sequences, with the only difference found in the control region. We found similar patterns other genera of the family Bibionidae – for instance, between *Bibiomarci* (OU343120.2; 16,014 bp) and *Bibiorufiventris* (MZ562678.1; 14,717 bp), and between the reference mitogenome of *Dilophusfebrilis* (OY284474.1; 19,009 bp) and its partial genome assembly (MT872668.1; 15,236 bp) – in which the differences in length are specifically due to shorter control regions. In all three cases, the larger genome assemblies were based on long-read sequence data, suggesting the superiority of long-read data in capturing control regions rich in tandem repeats compared to the Illumina short-read data, consistent with the previous observation in rhinoceros beetles (Morgan et al. 2022).

### ﻿Phylogenetic accounts

Our final concatenated supermatrix consisted of 14,192 bp nucleotide positions for 27 taxa, with the objectives of elucidating the relationships among the three subfamilies of Bibionidae. Within Bibionidae, the genera *Bibio*, *Dilophus*, *Penthetria*, and *Plecia* are represented by at least one complete mitogenome sequence (Tables 1, 2); however, due to the lack of mitogenomic sequence for the genus *Hesperinus*, we incorporated partial sequence data of three mitochondrial genes (12S, 16S rRNAs, and COI). For outgroup taxa, we included *Tipulaaestiva* (Diptera, Tipulomorpha, Tipulidae) as the most distant outgroup, and three brachyceran species for testing the monophyly of Bibionomorpha*sensu lato* (i.e., Anisopodoidea, Bibionoidea, Scatopsoidea, and Sciaroidea), as well as for addressing the persisting debate about the phylogenetic placements of Anisopodidae and Scatopsidae.

**Figure 2.**
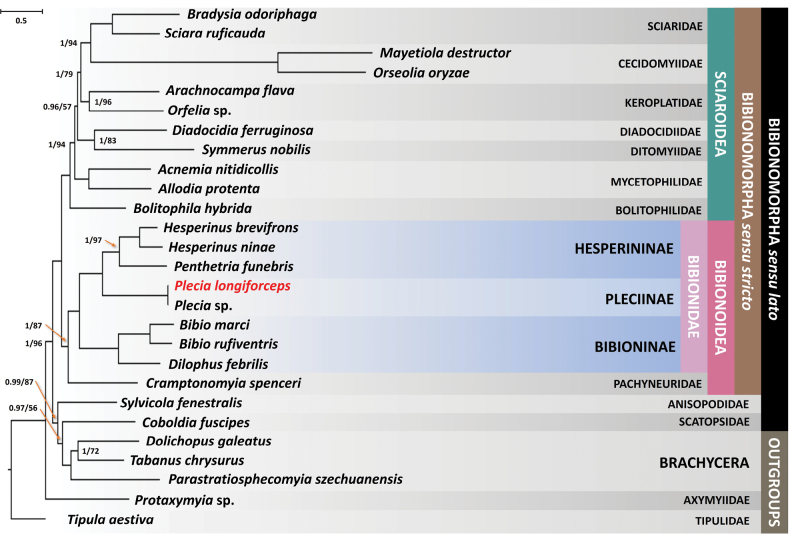
Bibionomorpha phylogeny inferred from the Bayesian analysis. Inferred phylogeny utilizing Bayesian analysis based on a matrix comprising 13 protein-coding genes and two ribosomal RNA genes originated from mitochondrial genomes, in total 14,192 base pairs. The analysis involved 25 taxa and included partial sequences of 12S, 16S ribosomal RNA genes, and mitochondrial cytochrome *c* oxidase subunit I (COI) from two *Hesperinus* species (*H.brevifrons* and *H.ninae*). Nodal values refer to posterior probability/ Ultrafast bootstrap support, respectively, are provided only for nodes lacking full support. The species highlighted in red is the newly sequenced mitochondrial genome in this study.

Despite the limited data for *Hesperinus*, both our ML and BI analyses produced congruent phylogenies across all nodes (Fig. 2). Along with the monotypy of the subfamily Pleciinae, the relationships within Bibionoidea – the family Pachyneuridae as a sister to Bibionidae, and Bibioninae as a sister group to Hesperininae and Pleciinae at the family- and subfamily-levels, respectively – were consistent with the previously reported phylogeny of Bibionomorpha (Ševčík et al. 2016). However, the monophyly of the subfamily Hesperininae (*Hesperinus* + *Penthetria*) was only marginally supported in our ML analysis, possibly due to the lack of complete mitogenomic sequences for the two *Hesperinus* species analyzed. Notably, the tip branch lengths of *Plecialongiforceps* and *Plecia* sp. (MZ562680.1) were found to be zero in both resulting trees, suggesting that these two species are potentially identical. However, further information about the specimen of *Plecia* sp. (MZ562680.1) is required to confirm this observation.

Our mitogenomic phylogeny of Bibionomorpha recovered the genus *Hesperinus* as one of the most derived lineages within Bibionidae, which is consistent with a previous molecular study that supported the inclusion of *Hesperinus* in Bibionidae and highlighted its close relationship with the genus *Penthetria* (Ševčík et al. 2016). While the sister relationship between *Hesperinus* and *Penthetria* was also supported in a morphology-based systematic study, the *Hesperinus* + *Penthetria* clade (or so-called Hesperininae) was suggested to represent the earliest-branching lineage within Bibionidae (Skartveit and Ansorge 2020). This basal placement of Hesperininae aligns with a historical view, which treated *Hesperinus* as a primitive and relictual lineage within Bibionomorpha closely related to Pachyneuridae (Krivosheina 1997). *Hesperinus* presents unique morphological characters, such as the sexually dimorphic antennae, that are commonly found in other primitive lineages of Bibionomorpha, including the families Bolitophilidae and Pachyneuridae (Fitzgerald 2004), as well as the fossil subfamily †Burmahesperininae (Bibionidae) (Ševčík et al. 2021). While the presence of elongated antennae even in the fossil species, †*Hesperinuselectrus* Skartveit, 2009, as well as the fossil subfamily †Burmahesperininae, could be indicative of their ancient origin and basal placement within Bibionidae, there remains a possibility that the sexually dimorphic, elongated antennae and other unique morphological features of *Hesperinus* represent homoplastic characters that resulted from specialized adaptations.

In fact, the species of *Hesperinus* present unique biology that clearly differentiates them from other bibionid flies. First, the larvae of *Hesperinus* develop inside decaying woods (Krivosheina 1997) and are not engaged in mass aggregation and migration in soil. Their life history inside decaying woods presumably resulted in a simplified soft body with sclerotized spiracles, similar to those of many wood-boring beetles (Chiappini and Aldini 2011), and the loss of the fleshy protuberances, which is a plesiomorphic character commonly found in other bibionids (Fitzgerald 2004). Such simplified traits may represent specialized adaptations to living in secure habitats with a limited need for locomotion. Moreover, the low density of larvae of *Hesperinus* in deciduous forest habitats are thought to have resulted in the absence of adult mating swarms, thereby requiring adult flies to rely on chemical signals over visual cues during their mate searching. Given that elongated antennae in male dipterans are thought to be associated with enhanced detection of pheromones (Vockeroth 1974), it is plausible that the long and sexually dimorphic antennae in *Hesperinus* is a character secondarily derived in this lineage, rather than an ancient morphology suggestive of its basal systematic placement. Moreover, the sexually non-dimorphic, dichoptic compound eyes of *Hesperinus* represents a character rarely found in other Bibionidae, whereby sexually dimorphic compound eyes are thought to be associated with visual recognition of females from the other males in mating swarms (Zeil 1983a, b). Taken together, despite the historical view of *Hesperinus* being the earliest branching lineage of Bibionidae, our phylogenetic analysis results, together with the discussion on their unique morphology being specialized adaptive traits, supports their derived placement within Bibionidae, sister to *Penthetria*.

While the test of phylogenetic relationships within the family Bibionidae was the primary focus of our study, our results allow a brief discussion on some of the strongly supported higher-level relationships within Bibionomorpha. The Bibionomorpha*sensu stricto* was recovered to consist of two distinct superfamilies – Bibionoidea and Sciaroidea – with monophylies of most of the currently recognized families within Bibionomorpha showing strong supports in all analyses. Within the superfamily Bibionoidea, the family Pachyneuridae, currently recognized by five extant species worldwide, was recovered as the earliest-branching lineage [BI posterior probability (BPP) = 1.0; ML bootstrap (MLB) = 87%], which was followed by the members of the monophyletic family Bibionidae (BPP = 1.0; MLB = 100%).

The monophyly of the Bibionomorpha*sensu lato* was not supported in our analyses, with the bibionomorphan families Anisopodidae and Scatopsidae being recovered to be more closely related to the suborder Brachycera with relatively strong supports (BPP = 0.99; MLB = 87%) (Fig. 2). Hennig (1981) originally hypothesized that Bibionomorpha*sensu lato* is a monophyletic group and sister to Brachycera, a relationship supported by multiple subsequent molecular studies (Bertone et al. 2008; Wiegmann et al. 2011; Ševčík et al. 2016). However, Oosterbroek and Courtney (1995) considered Anisopodidae to be distant from Bibionomorpha and placed it as a sister to Brachycera, a relationship congruent with the recent mitochondrial phylogeny by Zhang et al. (2023). Given the ongoing debate on the placement of Anisopodidae and Scatopsidae in Bibionomorpha*sensu lato*, as well as our finding of their potential affinity with Brachycera, a more comprehensive mitogenomic sampling of the two families and Brachycera is warranted to resolve their systematic positions within Diptera.

### ﻿Mass outbreaks in Bibionidae

Mass swarming behavior is a notable phenomenon observed in many bibionomorphan and other nematoceran species, whereby adult flies emerge in mass and often engage in mating swarms under various environmental conditions. Our study of a bibionid phylogeny based on mitochondrial genome data included at least one species from each of the three subfamilies – Hesperininae, Pleciinae, and Bibioninae – among which the representatives of Bibioninae and Pleciinae (e.g., *Bibiomarci*, *Dilophusfebrilis*, and *Plecialongiforceps*) are famously known for their mass outbreak behaviors. In particular, *Plecialongiforceps* was recently reported engage in mass outbreak in temperate Korea, following its range expansion from subtropical southeast China, similar to the classic case of *P.nearctica* in the southeastern United States (Kim et al. 2022). In addition to the two species of *Plecia*, the larvae of the Window gnat, *Sylvicolafenestralis* (Diptera, Anisopodidae), are known to develop in sewage sludge in urban areas and in decaying plant matter in natural habitats. These conditions lead to mass swarms, which can cause significant public nuisance (Coombs et al. 1997). Another extreme case is found in the family Trichoceridae (Tipulomorpha), commonly referred to as “winter crane flies”, which prefer cold temperatures and are known to form swarms on snowy surfaces. For instance, *Trichoceramaculipennis* Meigen, 1818, was recently reported to have been introduced anthropogenically from the Northern Hemisphere to ice-free areas of Antarctica, where they are engaged in mass outbreak (Potocka and Krzemińska 2018).

Given the continued range expansion of swarming dipteran species driven by global climate change and anthropogenic influences, as well as their potentially aggravating impact in introduced regions, understanding the biological implications of mass outbreak behavior in Diptera has become critical. One of the most common and significant traits among bibionomorphan flies is the detritivorous feeding habit of their soil-dwelling larvae, which often aggregate in large numbers in humid soil environments under low temperature and desiccation stress. In these species, ecological stressors are known to induce phenotypic plasticity within their life cycles, modulating the timing of adult emergence. This adaptive response synchronizes their swarming outbreaks upon optimal environmental conditions (Krivosheina et al. 2019). Accordingly, *P.longiforceps* exhibits a univoltine lifecycle in temperate Korea with a single adult emergence in early summer (Kim et al. 2022), while they are known to be bivoltine in their native subtropical habitats (Duda 1933). This observation highlights the exceptional plasticity of these dipteran species to changing climate conditions, which likely is associated with their larval aggregation behavior and subsequent adult emergence in mass. Another dipteran species in the family Psychodidae, *Pericomablandula* Eaton, 1893, also develops in larval aggregates, and display various life cycles across different climate zones (e.g., univoltine in subarctic Scandinavia, bivoltine in temperate Central Europe, and polyvoltine in the Mediterranean Region) (Wagner 1997).

While there seems to be an apparent link between the ecological plasticity of swarming behavior of bibionomorphan species and their adaptive ability to changing climate conditions, the extent and evolutionary implication of mass outbreak behavior remain to be tested based on a comprehensive phylogenomic study with improved taxon sampling that reflects the breadth of diverse ecological traits observed even among closely related species. For example, *Pleciaamericana*, whose distribution overlaps with *P.nearctica* in the United States, is not involved in mass outbreak and is typically found in woodland habitats in small quantities (Hardy 1940; Buschman 1976). This contrasts sharply with the mass outbreak behavior observed in *P.nearctica* and *P.longiforceps*. Our newly sequenced complete mitochondrial genome of *P.longiforceps* contributes to an ongoing phylogenomic investigation of Bibionomorpha, which, despite being far from complete, opens an inquiry into the evolutionary origin of mass outbreak and their adaptive competency in the face of global climate change.

## ﻿Additional information

### Author ORCIDs

Seunghun Jung https://orcid.org/0000-0002-8065-6696

Sangil Kim https://orcid.org/0000-0003-2758-8943

Seunggwan Shin https://orcid.org/0000-0002-4258-8661

### Data availability

The complete mitochondrial genome sequence data of *Plecialongiforceps* are openly available in GenBank under the accession number PP060435.
